# Reply to: Uncertain climate effects of anthropogenic reactive nitrogen

**DOI:** 10.1038/s41586-025-09338-8

**Published:** 2025-10-22

**Authors:** Cheng Gong, Hanqin Tian, Hong Liao, Naiqing Pan, Shufen Pan, Akihiko Ito, Atul K. Jain, Sian Kou-Giesbrecht, Fortunat Joos, Qing Sun, Hao Shi, Nicolas Vuichard, Qing Zhu, Changhui Peng, Federico Maggi, Fiona H. M. Tang, Sönke Zaehle

**Affiliations:** 1 Max Planck Institute for Biogeochemistry, Jena, Germany; 2 Center for Earth System Science and Global Sustainability, Schiller Institute for Integrated Science and Society, Boston College, Chestnut Hill, MA USA; 3 Department of Earth and Environmental Sciences, Boston College, Chestnut Hill, MA USA; 4 School of Environmental Science and Engineering, Nanjing University of Information Science and Technology, Nanjing, China; 5 International Center for Climate and Global Change Research, College of Forestry, Wildlife and Environment, Auburn University, Auburn, AL USA; 6 Department of Engineering and Environmental Studies Program, Boston College, Chestnut Hill, MA USA; 7 Graduate School of Agricultural and Life Sciences, University of Tokyo, Tokyo, Japan; 8 Earth System Division, National Institute for Environmental Studies, Tsukuba, Japan; 9 Department of Atmospheric Science, University of Illinois, Urbana-Champaign, Urbana, IL USA; 10 Department of Earth and Environmental Sciences, Dalhousie University, Halifax, Nova Scotia Canada; 11 Climate and Environmental Physics, Physics Institute, University of Bern, Bern, Switzerland; 12 Oeschger Centre for Climate Change Research, University of Bern, Bern, Switzerland; 13 State Key Laboratory of Urban and Regional Ecology, Research Center for Eco-Environmental Sciences, Chinese Academy of Sciences, Beijing, China; 14 Laboratoire des Sciences du Climat et de l’Environnement, LSCE-IPSL (CEA-CNRS-UVSQ), Université Paris-Saclay, Gif-sur-Yvette, France; 15 Climate and Ecosystem Sciences Division, Lawrence Berkeley National Lab, Berkeley, CA USA; 16 Department of Biology Sciences, Institute of Environment Science, University of Quebec at Montreal, Montreal, Quebec Canada; 17 School of Geographic Sciences, Hunan Normal University, Changsha, China; 18 Environmental Engineering, School of Civil Engineering, The University of Sydney, Sydney, New South Wales Australia; 19 Department of Civil Engineering, Monash University, Clayton, Victoria Australia

**Keywords:** Element cycles, Atmospheric chemistry

replying to: Ø. Hodnebrog et al. *Nature* 10.1038/s41586-025-09337-9 (2025).

The main purpose of Gong et al.^1^ is to show that anthropogenic reactive nitrogen (Nr) has a net cooling influence on climate, which has important implications for future emissions mitigation strategies. We welcome that in the accompanying Comment^2^, Hodnebrog et al. confirm the net cooling influence of anthropogenic Nr. However, Hodnebrog et al. argue that Gong et al. underestimate the uncertainties in individual effects, such as in aerosol, ozone (O_3_) and methane (CH_4_) radiative forcing (RF) from Nr emissions. Here we show that the varied differences of each component will not influence the estimates of the net climate effect under future projections, and we find that biases and uncertainties in Hodnebrog overemphasize differences between our and their estimates.

**Fig. 1:**
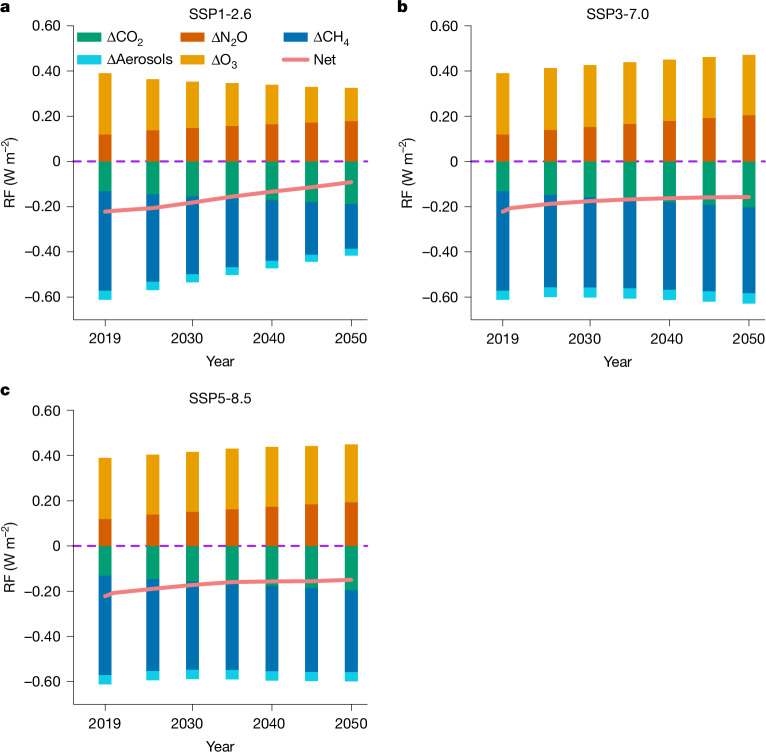
Prediction of the climate effects of anthropogenic Nr to 2050. The present-day RFs of each component are following ‘the central estimates’ in Hodnebrog et al.^2^, which are −0.13 W m^−2^ of CO_2_, +0.12 W m^−2^ of N_2_O, −0.44 W m^−2^ of CH_4_, −0.04 W m^−2^ of aerosols and +0.27 W m^−2^ of O_3_. The predicted climate effects of anthropogenic Nr are following the scenarios of SSP 1-2.6 (**a**), SSP 3-7.0 (**b**) and SSP 5-8.5 (**c**). The cascading effects of CH_4_ changes on tropospheric O_3_ and stratospheric water vapour are included here following Hodnebrog et al.^2^ but excluded in Gong et al.^1^. The rest of the method is identical to that in Fig. 5 in Gong et al.^1^. SSP, Shared Socioeconomic Pathway.

Although we disagree that the central estimates in Hodnebrog et al. are comparable to those of Gong et al.^1^ (see below), we first apply their central estimates of each component to estimate the sensitivities of RF to carbon dioxide (CO_2_), nitrous oxide (N_2_O), CH_4_ concentrations, or ammonia (NH_3_) and nitrogen oxide (NO_*x*_) emissions, respectively, and reproduce the assessment of future impacts (Fig. 5 in Gong et al.^1^). Figure 1 shows that this update provides similar patterns (in terms of magnitude and trend across the three scenarios) in RF change as in Gong et al., where the differences between the updated and original trends are fully covered by the original uncertainty ranges in Gong et al. We acknowledge that these future estimates are based on simple calculations and, as already written in Gong et al., we encourage further work to integrate more dynamic feedbacks into future projections of the net climate effect of anthropogenic Nr. However, our new analysis indicates that the associated uncertainties will not “carry important implications for future projections” as Hodnebrog et al. argued.

An important uncertainty in Hodnebrog et al.^2^ is that the RFs of aerosols and O_3_ are not calculated by the online radiative transfer modules in each chemistry–climate model, but by prescribed monthly three-dimensional maps of aerosol and O_3_ kernel ‘radiative efficiency’ (united by Watts per gram change in aerosol loading (W/g) or Watts per Dobson unit change in O_3_ (W/DU)) generated from OsloCTM3. Such a simplified method fails to account for the inter-model differences in the particle physical properties (for example, sizes, humidity and mixture), cloudiness distributions and surface albedo, all of which have very high temporal heterogeneity and thus introduce uncertainty into the assessment of the short-lived greenhouse components aerosol and O_3_.

Hodnebrog et al.^2^ argue that aerosol cooling effect induced by anthropogenic Nr is substantially weaker than that in Gong et al.^1^. Although we have explicitly acknowledged in the main text that “the negative radiative forcing of nitrate aerosol may be overestimated, as the GEOS-Chem model tends to overestimate nitrate aerosol concentrations”^3–5^, we find that the enhancements of fine-mode nitrate loadings in CESM2 (0.068 Tg yr^−1^) and OsloCTM3 (0.089 Tg yr^−1^) are also at the low end relative to the ranges given by AeroCom III multi-models^6^. The positive sulfate aerosol RFs in GISS-MATRIX and OsloCTM3 are also questionable and require more validation. Furthermore, Hodnebrog et al. assume all sulfate exists in the form of ammonium sulfate ((NH_4_)_2_SO_4_) when calculating RF, which may enhance global pre-industrial aerosol mass in the No_allNr experiment and further weaken the present-day aerosol RF, as the dominant sulfate phase under an ammonia-poor environment (for example, in the form of H_2_SO_4_) has lower molecular weight than (NH_4_)_2_SO_4_.

We are also concerned about the result of the simplified method applied by Hodnebrog et al.^2^ to derive changes in CH_4_ concentration from NO_*x*_ emissions. The implied lifetime changes of CH_4_ for a change in NO_*x*_ loading, derived from inverting the calculation of CH_4_ concentration in Hodnebrog et al. (see their methods) suggests that NO_*x*_ reduces CH_4_ lifetime in the GISS-Matrix model by approximately 50%. This is clearly outside the range of a 22–34% reduction in CH_4_ lifetime as a result of the NO_*x*_ emission changes between 1850 and 2000 using a multi-model ensemble^7^. The other models, including our own estimates, are either at the upper (CESM, LMDZ) or lower (OsloCTM, GFDL, as well as our own estimate) end of this range. This finding is also consistent with the NO_*x*_-induced forcing due to CH_4_-lifetime changes in the multi-model ensemble in ref. ^8^ (−0.2 W m^−2^ to −0.37 W m^−2^), which identifies the GISS-MATRIX model used in Hodnebrog et al. as an extreme outlier (−0.53 W m^−2^) for GISS-MATRIX)), whereas the CESM and LMDZ are at the high end. The additional effects considered by Hodnebrog et al. but not in Gong et al.^1^ — that is, CH_4_ impacts on tropospheric O_3_ and stratospheric water — slightly affect our mean estimate, but remain within the uncertainty range provided in the original paper.

The RF of O_3_ induced by anthropogenic Nr in Gong et al.^1^ (+0.03 W m^−2^ to +0.07 W m^−2^) is at the lower end boundary of the Intergovernmental Panel on Climate Change Sixth Assessment Report model ensemble^8,9^ (+0.07 W m^−2^ to +0.27 W m^−2^), which we have already noted in Supplementary Table 3 in ref. ^1^, relative to ref. ^8^ with a range of 0.2 ± 0.07 W m^−2^. The comparison brought up by Hodnebrog et al.^2^ therefore provides no new information regarding the wide across-model variations in the O_3_ RF induced by anthropogenic NO_*x*_ emissions. We note that the estimates of CESM2, GISS-MATRIX and LMDZ-INCA (around +0.3 W m^−2^ to +0.35 W m^−2^) exceed the upper boundary of ref. ^8^, which contributes to exaggerating the differences between our results and ref. ^8^.

We agree that line-by-line radiative transfer calculations provide the most accurate estimates. However, GEOS-Chem RRTMG is internally consistent in Gong et al.^1^ and includes broadband treatment between the different forcing factors. We note that the differences in N_2_O and CO_2_ will not significantly change the net climate effects as well as the future projections in our study.

Last but not least, we argue that the accuracy of model predictions should ideally not be determined by the uncertainty ranges of multi-model means, but by their evaluation against observations. Hodnebrog et al. do not cite any evidence that these five models have better performance compared against observations than GEOS-Chem. In particular, four of the five models are climate–chemistry models, and their simulations are affected by uncertainties in meteorology simulations, simplified chemical mechanisms and intricate feedback mechanisms^10–12^. In contrast, the GEOS-Chem model has been widely evaluated across different continents against surface observations, aircraft campaigns and satellite retrievals (for example, refs. ^3,13–16^).

We acknowledge that the RF values of specific Nr components are subject to uncertainty resulting from using more ensemble members with higher degrees of feedback processes, but the dominant processes associated with the climate effects of anthropogenic Nr have been properly addressed in Gong et al.^1^.
